# Functional expression of an oxygen-labile nitrogenase in an oxygenic photosynthetic organism

**DOI:** 10.1038/s41598-018-25396-7

**Published:** 2018-05-09

**Authors:** Ryoma Tsujimoto, Hiroya Kotani, Konomi Yokomizo, Hisanori Yamakawa, Aoi Nonaka, Yuichi Fujita

**Affiliations:** 1 Graduate School of Bioagricultural Sciences, Nagoya University, Nagoya, 464-8601 Japan; 2 School of Agricultural Sciences, Nagoya University, Nagoya, 464-8601 Japan

## Abstract

Transfer of nitrogen fixation ability to plants, especially crops, is a promising approach to mitigate dependence on chemical nitrogen fertilizer and alleviate environmental pollution caused by nitrogen fertilizer run-off. However, the need to transfer a large number of nitrogen fixation (*nif*) genes and the extreme vulnerability of nitrogenase to oxygen constitute major obstacles for transfer of nitrogen-fixing ability to plants. Here we demonstrate functional expression of a cyanobacterial nitrogenase in the non-diazotrophic cyanobacterium *Synechocystis* sp. PCC 6803 (*Synechocystis* 6803). A 20.8-kb chromosomal fragment containing 25 *nif* and *nif*-related genes of the diazotrophic cyanobacterium *Leptolyngbya boryana* was integrated into a neutral genome site of *Synechocystis* 6803 by five-step homologous recombination together with the *cnfR* gene encoding the transcriptional activator of the *nif* genes to isolate CN1. In addition, two other transformants CN2 and CN3 carrying additional one and four genes, respectively, were isolated from CN1. Low but significant nitrogenase activity was detected in all transformants. This is the first example of nitrogenase activity detected in non-diazotrophic photosynthetic organisms. These strains provide valuable platforms to investigate unknown factors that enable nitrogen-fixing growth of non-diazotrophic photosynthetic organisms, including plants.

## Introduction

Nitrogen fixation is the conversion of molecular nitrogen (N_2_) to ammonia, a more accessible nitrogen source for most organisms. Biological nitrogen fixation is catalyzed by nitrogenase, which can be classified into three isoforms according to metal contents in the catalytic components: MoFe-nitrogenase^1^, VFe-nitrogenase^2,3^, and FeFe-nitrogenase^4,5^. MoFe-nitrogenase has been most extensively studied^6–8^, and the reaction catalyzed by MoFe-nitrogenase is as follows:

$${{\rm{N}}}_{2}{+8{\rm{H}}}^{+}{+8{\rm{e}}}^{{\textstyle -}}+16{\rm{A}}{\rm{T}}{\rm{P}}+16{{\rm{H}}}_{2}{\rm{O}}\to {2{\rm{N}}{\rm{H}}}_{3}+{{\rm{H}}}_{2}+16{\rm{A}}{\rm{D}}{\rm{P}}+16{\rm{P}}{\rm{i}}$$

MoFe-nitrogenase (hereafter called just nitrogenase) consists of two components, Fe protein and MoFe protein. The Fe protein, a homodimer of NifH, acts as an ATP-dependent reductase for the catalytic component MoFe protein by accepting electrons from ferredoxin or flavodoxin (dithionite for *in vitro* systems) and transferring them to MoFe protein in a process coupled to ATP hydrolysis. MoFe protein, a heterotetramer of NifD and NifK, serves as the catalytic component. The MoFe protein contains two metallocenters, P-cluster and FeMo-cofactor (FeMo-co). Electrons from the Fe protein are transferred to FeMo-co via P-cluster and dinitrogen bound to the FeMo-co is reduced to ammonia. The three metallocenters required for nitrogen fixation, the [4Fe-4S] cluster of Fe protein, the P-cluster, and the FeMo-co, are extremely vulnerable to oxygen with half-lives of seconds to minutes upon exposure to the air^9^. In addition, biosynthesis of P-cluster and FeMo-co require many *nif* gene products (at least eight; *nifHBSUENVZ*)^10^, and like the metallocenters of nitrogenase, precursors of FeMo-co such as NifB-cofactor are very sensitive to oxygen. Thus, an anaerobic environment is necessary for biosynthesis and operation of nitrogenase.

Nitrogen supply is one of the main factors determining crop yield. Industrial nitrogen fixation, invented at the beginning of the 20^th^ century, has allowed for the large-scale production of chemical nitrogen fertilizer from the atmosphere, which in turn has markedly increased crop yields during the past century^11^. However, industrial nitrogen fixation consumes massive amounts of fossil fuel, contributing to the increase in atmospheric CO_2_. Furthermore, significant amounts of nitrogen fertilizer leak into the environment due to excess application and inefficient assimilation into crops, resulting in serious environmental pollution from reactive nitrogen species^12,13^. Conferring crops with nitrogen fixing ability is one promising technology for alleviating these deleterious environmental consequences. One such approach is the transfer of genes encoding nitrogenase directly to the crop genome^14,15^. Transgenic crops expressing active nitrogenase could then convert atmospheric nitrogen to ammonia as an *in situ* nitrogen source.

However, there are two major obstacles to this “new agriculture using the air as nitrogen fertilizer”. One obstacle is the oxygen vulnerability of nitrogenase and enzymes involved in biosynthesis of nitrogenase metallocenters. Crops are photosynthetic organisms that produce oxygen by photosynthesis. Thus, nitrogenase must be protected not only from atmospheric oxygen but also from endogenously produced oxygen in plants. The other obstacle is the required co-expression of at least 9 *nif* genes for nitrogenase and for biosynthesis of the metalloclusters at appropriate levels^16^. In addition, abundant reducing power (reduced ferredoxin) and ATP are required for nitrogenase (see reaction formula above). Mitochondria and chloroplasts (plastids) have been proposed as cellular compartments to accommodate active nitrogenase in plant cells^14,17^. The mitochondrial matrix provides a low oxygen environment due to active respiration at the inner membranes. However, the mitochondrial ferredoxin (adorenodoxin) does not serve as an electron donor to nitrogenase^18^. Alternatively, chloroplasts produce enough reduced ferredoxin and ATP by active photosynthetic electron transfer in thylakoid membranes under light conditions. Moreover, endogenous ferredoxins are able to serve as electron donors to nitrogenase^18^. On the other hand, oxygen production by photosystem II may be incompatible with oxygen-sensitive nitrogenase. Chloroplast genomes of many algae and plants such as gymnosperms (e.g., *Pinus thunbergii*)^19^ and mosses (e.g., *Physcomitrella patens* and *Marchantia polymorpha*)^20,21^ encode three subunits of a nitrogenase-like enzyme, dark-operative protochlorophyllide oxidoreductase (DPOR), that shows similar oxygen sensitivity as nitrogenase^20^. This suggests that chloroplasts have the potential ability to express and accommodate active nitrogenase^14^.

Cyanobacteria are prokaryotes that perform oxygenic photosynthesis similar to plants and share a common evolutionary ancestor with chloroplasts. About half of cyanobacterial species have the ability of nitrogen fixation^22^. In some of these species, the coexistence of oxygenic photosynthesis and nitrogen fixation is achieved by spatial separation in differentiated cells specialized for nitrogen fixation termed heterocysts (heterocystous cyanobacteria)^23^. For non-heterocystous cyanobacteria, it has been proposed that photosynthesis and nitrogen fixation are separated temporally under control of the circadian clocks^24,25^. However, some non-heterocystous cyanobacteria show nitrogenase activity only under light conditions^26–28^, implying the existence of some molecular mechanisms to solve the “oxygen paradox” between oxygen-sensitive nitrogenase and oxygen-producing photosynthesis.

Cyanobacteria without nitrogen fixing ability could serve as a suitable model acceptor for genes involved in nitrogen fixation. In this study, we isolated a transformant CN1 of the non-diazotrophic cyanobacterium *Synechocystis* sp. PCC 6803 (*Synechocystis* 6803) harboring a long *nif* gene cluster (20.8 kb) from the diazotrophic cyanobacterium *Leptolyngbya boryana* as a model experiment for transplantation of *nif* genes in non-diazotrophic photosynthetic organisms. In addition, two other transformants CN2 and CN3 were also isolated from CN1. All three transformants, CN1, CN2 and CN3 carrying 26, 27 and 30 *nif* and *nif*-related genes, respectively, exhibited low but significant nitrogenase activity. This is the first example of functional nitrogenase expression in an oxygenic photosynthetic organism. These transformants provide promising platforms to identify factors allowing nitrogen fixation in oxygenic photosynthetic organisms and to improve nitrogen fixing growth in non-diazotrophic photosynthetic organisms.

## Results

### Step-wise integration of the *nif* gene cluster into the chromosome of *Synechocystis* 6803

**Figure 1:**
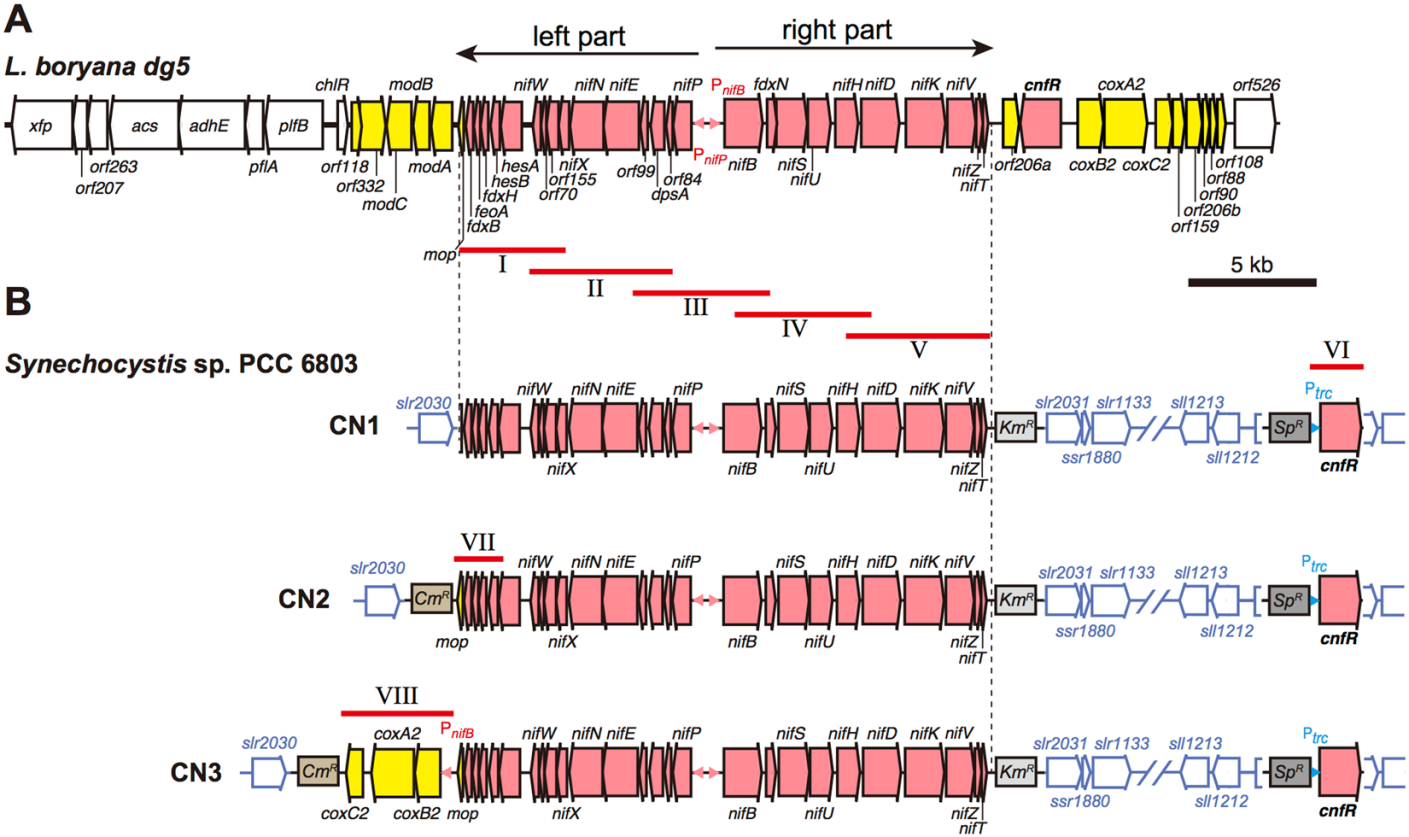
(**A**) The 50-kb *nif* gene cluster in *L. boryana dg5*. Genes that were transferred to the genome of *Synechocystis* 6803 to isolate CN1 are shown in pink. Genes in this central region (shown in two arrows of right and left parts) are divergently transcribed from the *nifB* (P_*nifB*_) and *nifP* (P_*nifP*_) promoters (shown by small pink triangles). Expression of all these genes except *cnfR* is dependent on CnfR^29^. Genes denoted in yellow show the highest transcript levels under nitrogen fixing conditions and their transcripts are still detected in the absence of *cnfR* (*∆cnfR*), albeit lower than wild type^29^. Subfragments I–V introduced sequentially into the genome neutral site III are shown by red lines. (**B**) Gene arrangements of the transferred *nif* gene cluster in *Synechocystis* 6803 transformants CN1, CN2, and CN3. Subfragments VI, VII, and VIII (red lines) were used for isolation of CN1, CN2, and CN3, respectively.

In a previous study, we identified the *cnfR* gene encoding the transcriptional activator for *nif* genes in the 50-kb gene cluster of the *L. boryana* chromosome^29^. All *nif* and *nif*-related genes activated by CnfR are concentrated in the central 20-kb region of the gene cluster. In this region, there are 25 genes containing the minimal gene repertory to produce active nitrogenase components Fe protein and MoFe protein^16^ (Fig. 1A): 14 *nif* genes (*nifBSUHDKVZT* in the right part and *nifPENXW* in the left part), 5 *nif*-related genes (*fdxB*, *fdxH*, *hesAB* and *fdxN*), and 6 genes with unknown functions (*feoA*, *orf70*, *orf155*, *orf90*, *dpsA*, and *orf84*). These genes are divergently transcribed from the intergenic region between *nifB* and *nifP* upon activation by CnfR^29,30^. We also confirmed that this activation system operates in *Synechocystis* 6803^30^. We then examined whether nitrogen fixation can be conferred to *Synechocystis* 6803 by introduction of the 20-kb *nif* gene cluster together with the *cnfR* gene into its chromosome.

*Synechocystis* 6803 is naturally transformable, and exogenous DNA fragments are incorporated and integrated into the chromosome via homologous recombination. However, the >20-kb fragment needed for transfer of nitrogen fixation ability would be difficult to integrate stably in a single step. Thus, the 20-kb DNA fragment was divided into five subfragments (Fragments I to V; Fig. 1) with some overlap and integrated into a neutral site of the acceptor chromosome in 5 steps (Supplementary Figs 1 and 2).

The first fragment (Fragment I) was introduced into neutral site III (between *slr2030-slr2031*) along with the kanamycin resistance gene (Km^R^) as a selection marker to isolate the first transformant SN1 (Supplementary Fig. 2). In SN1, 8 genes and a 3′ region of *nifX* were integrated. The second fragment (Fragment II) was introduced between Fragment I and *slr2031* of SN1 with the spectinomycin resistance gene (Sp^R^) as an alternative selection marker to isolate SN2. The third fragment (Fragment III) was integrated between Fragment II and *slr2031* by Km^R^ selection to isolate SN3. The fourth fragment (Fragment IV) was integrated between Fragment III and *slr2031* by Sp^R^ selection to isolate SN4. Finally the fifth fragment (Fragment V) was integrated between Fragment IV and *slr2031* by Km^R^ selection to isolate SN5. In SN5, the 20.8-kb (20,763 bp) fragment containing 25 genes (from *fdxB* to *nifT*) and the Km^R^ gene (2,230 bp) was inserted between *slr2030* and *slr2031*.

### Isolation of CN1, CN2, and CN3

A chimeric fragment (Fragment VI) in which the *cnfR* gene was connected to the *trc* promoter (derived from pQE-70) and the selective marker Sp^R^ was integrated into neutral site I, the coding region of *psbA2* (*slr1311*) in SN5, to isolate CN1.

In SN5 and CN1, a 5′ region of the *mop* gene was introduced downstream of the leftmost *nif* gene cluster gene *fdxB*. Another transformant with intact *mop* gene, CN2, was isolated by integration of the seventh fragment (Fragment VII) between *slr2030* and *fdxB* using the chloramphenicol resistance gene (Cm^R^) as the selection marker (Fig. 1B, Supplementary Figs 1 and 2). In CN2, the 20.9-kb (20,909-bp) fragment containing the 26 genes from *mop* to *nifT*, the Cm^R^ gene (1,599 bp), and the Km^R^ gene (2,230 bp) was inserted between *slr2030* and *slr2031*.

The *cox* genes (*coxB2*, *coxA2*, and *coxC2*) located just downstream of *cnfR* in the *nif* gene cluster in *L. boryana* encode subunits of cytochrome *c* oxidase (COX), and COX is probably involved in oxygen protection and ATP generation for nitrogenase. A chimeric fragment (Fragment VIII) of the truncated *nifB* promoter (484 bp), the *coxB2-coxA2-coxC2* gene cluster, and the intact *mop* gene was integrated between *slr2030* and *fdxB* in CN1 to yield CN3 (Fig. 1B, Supplementary Figs 1 and 2). In CN3, the 24.9-kb (24,863-bp) fragment containing 29 genes (from *mop* to *nifT*, and *coxB2* to *coxC2*), the Km^R^ gene (2,230 bp), and the Cm^R^ gene (1,599 bp) was inserted between *slr2030* and *slr2031*. We expected that the *cox* genes would be expressed by activation of CnfR and that the resultant COX complex would stimulate nitrogenase activity by supplying of ATP and removing residual oxygen.

Genome resequencing of CN1, CN2, and CN3 confirmed that all gene manipulations resulted as intended except for a single SNP, a T-to-C conversion causing one amino acid residue substitution, Phe150 to Leu, in the NifE protein. Alignment of various cyanobacterial NifE proteins from 30 species including *L. boryana* suggested that the residue at this site is not conserved (namely Ile, Val, or Phe), while the NifE protein from *Tripothrix campylonemoides* has Leu at this position. We concluded that this SNP would not strongly affect the activity of NifE for active nitrogenase production in these transformants.

### Nitrogenase activity in CN1, CN2, and CN3

**Figure 2:**
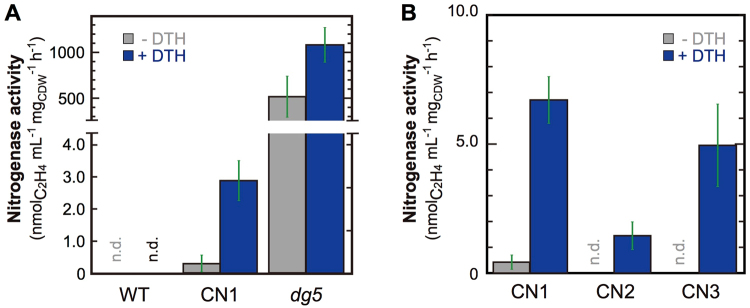
(**A**) Nitrogenase activity of *Synechocystis* 6803 WT, CN1, and *L. boryana dg5* as estimated by ethylene (C_2_H_4_) formation in the presence (blue) and absence (gray) of dithionite. Nitrogenase activity was estimated as ethylene formation per CDW. Incubation time was 2 h. (**B**) Nitrogenase activity of the three transformants CN1, CN2, and CN3. Incubation time was 5 h. Error bars (green) represent standard deviations (n = 3). n.d. denotes “not detected”.

We first examined whether CN1 expresses nitrogenase activity using the ethylene formation assay. Cells grown under aerobic and nitrate-replete conditions were shifted to micro-oxic and nitrogen-deficient conditions to induce expression of *nif* genes by CnfR, and ethylene formation from acetylene was measured under anaerobic and light conditions. To normalize cell density between two species, *L. boryana* and *Synechocystis* 6803, we estimated acetylene reduction activity per cell dry weight (CDW, Supplementary Fig. 3). Dithionite was added to remove oxygen produced endogenously by photosynthesis, thereby enhancing nitrogenase activity. Wild type (WT) *L. boryana* (*dg5*) cells showed ethylene production of about 500 (517 ± 226) and 1,000 (1,084 ± 190) nmol ml^−1^ h^−1^ mg_cdw_^−1^ in the absence and presence of dithionite, respectively (Fig. 2A), confirming that the addition of dithionite has a significant positive effect on *in vivo* nitrogenase activity as reported^31^. While no activity was detected in WT *Synechocystis* 6803, CN1 showed very low but significant ethylene formation in the presence of dithionite (2.8 ± 0.6 nmol ml^−1^ h^−1^ mg_cdw_^−1^, Fig. 2A and Supplementary Figs 4 and 5). The activity of CN1 was about 0.26% of that of *L. boryana* WT (in the presence of dithionite). Then, we compared activities of the three transformants, CN1, CN2 and CN3 (Fig. 2B). Activity of CN2 was significantly lower than that of CN1 (p < 0.05). CN3 showed comparable or even slightly lower nitrogenase activity to CN1 (p = 0.38). These results clearly indicate that a small but significant amount of active nitrogenase can be produced in all transformants.

We examined whether the three transformants CN1, CN2, and CN3 are able to grow under nitrogen fixing conditions. However, none of the *Synechocystis* 6803 transformants showed significant growth even under micro-oxic conditions for 23 days (Supplementary Fig. 6). This result suggests that nitrogen fixing ability was not conferred in these transformants despite integration of 25, 26, and 29 *nif* and *nif*-related genes.

### Estimation of nitrogenase proteins in CN1

**Figure 3:**
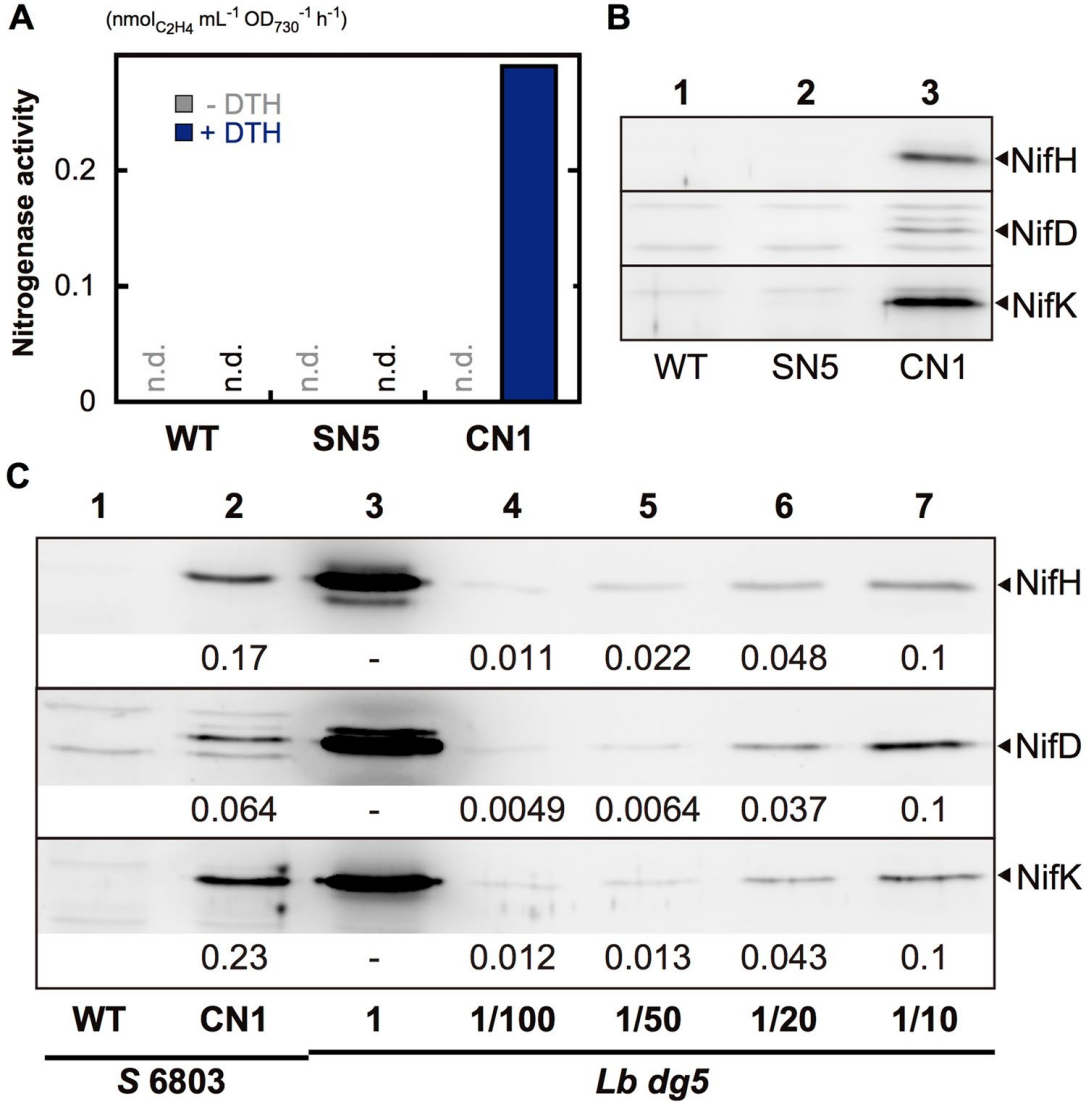
(**A**) Nitrogenase activity as measured by ethylene formation for WT, SN5, and CN1 *Synechocystis* 6803 in the presence (blue) and absence (gray) of dithionite. (**B**) Western blot analysis of crude extracts from WT (lane 1), SN5 (lane 2) and CN1 (lane 3) *Synechocystis* 6803 using antisera against NifH, NifD, and NifK. Five hundred nanograms of protein was loaded onto each lane. (**C**) Estimated expression levels of NifH, NifD, and NifK by CN1 (0.5 µg, lane 2) compared to whole crude extract of *L. boryana dg5* (0.5 µg, lane 3) and serial dilutions (10-fold dilution, lane 7; 20-fold dilution, lane 6; 50-fold dilution, lane 5; and 100-fold dilution, lane 4). Numbers below the signals are relative band intensities estimated by densitometry normalized to 0.1 for the band in 10-fold dilution (ImageJ 1.50i).

To examine the expression levels of *nif* genes in CN1 following CnfR induction, we conducted Western blot analysis of cell extracts using antisera against NifH, NifD, and NifK (Fig. 3). The direct parental transformant for CN1, SN5, does not harbor the *cnfR* gene and so was examined as a control. After treatment for nitrogenase induction as above, WT, SN5, and CN1 cells were subject to nitrogenase assay (Fig. 3A), and total cell extracts were prepared for Western blot analysis (Fig. 3B). Neither SN5 nor WT *Synechocystis* 6803 showed detectable activity, while CN1 showed significant activity as in Fig. 2. In Western blots of CN1 extracts demonstrated clear bands for NifH, NifD, and NifK, while no bands for any of these proteins were detected from SN5 extracts. This result supports our initial notion that *nifB* and *nifP* promoters can be activated by CnfR to produce *nifHDK* transcripts and NifHDK proteins in non-diazotrophic cyanobacterium.

Furthermore, we estimated NifHDK protein expression levels in CN1 compared to *L. boryana dg5* by Western blot analysis (Fig. 3C). The NifHDK band intensity from CN1 extract was similar to that produced by a 1/10 diluted extract of *L. boryana dg5* (compare lanes 2 and 7 in Fig. 3C). The intensities of the signals of NifH, NifD, and NifK bands from CN1 were 0.17, 0.064, and 0.23, respectively (lane 2, Fig. 3C). Considering that the ethylene formation activity of CN1 was about 0.26% (CDW-basis) of that of *L. boryana*, it is estimated that only 1.5% of total NifH, 4.1% of total NifD, and 1.1% of total NifK in CN1 are assembled into active nitrogenase constituents.

## Discussion

In this study we isolated three transformants of the non-diazotrophic cyanobacterium *Synechocystis* 6803, harboring 25 (strain CN1), 26 (CN2), and 29 (CN3) genes of the diazotrophic cyanobacterium *L. boryana*. All three CN strains showed a low but significant nitrogenase activity under micro-oxic and nitrogen-deficient conditions, indicating that CnfR activates the divergent transcription of *nifB* and *nifP* promoters in the non-diazotrophic heterologous host *Synechocystis* 6803. This result is consistent with the previous reporter experiments of the *nifB* and *nifP* promoters in *Synechocystis* 6803^30^. Although these transformants did not grow diazotrophically, this is the first successful example of heterologous active nitrogenase expression in an oxygenic photosynthetic organism. This result indicates that the 25 genes within the 20.8-kb fragment integrated into CN1 are sufficient for expression of active nitrogenase in oxygenic photosynthetic cells.

Although the expression levels of NifHDK proteins in CN1 were about 6.4%–23% of those in *L. boryana* (Fig. 3C), nitrogenase activity was only 0.26% that of *L. boryana* (Fig. 2A), suggesting that only small proportions (1.1%–4.1%) of expressed NifHDK proteins contribute to formation of active nitrogenase. This simple calculation may underestimate the proportions of active nitrogenase proteins in *Synechocystis* 6803. Even if the nitrogenase assembles normally in *Synechocystis* 6803 cells, its operating conditions may be suboptimal due to heterogeneous expression. Large amounts of reducing power and ATP should be supplied to support nitrogenase activity. The most efficient electron donor for cyanobacterial nitrogenase is a ferredoxin FdxH. The *fdxH* gene is contained in the *nif* gene cluster that was introduced into the CN strains. Thus, FdxH would donate electrons to nitrogenase in CN1. However, the endogenous ferredoxin PetF of *Synechocystis* 6803, which could be less efficient as an electron donor to nitrogenase^32^, may compete with FdxH in binding to nitrogenase during transferring electrons, resulting in lowering nitrogenase activity^33^. It is also likely that ATP production is not enough to support nitrogenase activity. In any case, it is inferred that the inability for diazotrophic growth by CN strains is due to the extremely low nitrogenase activity.

Furthermore, the low efficiency of NifHDK assembly is probably caused by the oxygen vulnerability of nitrogenase and assembly proteins, such as NifB, NifU and NifEN, involved in the biosynthesis of nitrogenase metal centers. *Synechocystis* 6803 has the ability to grow under micro-oxic conditions^34^, but the capacity to remove or consume oxygen may not be high enough for nitrogenase to assemble and operate under micro-oxic conditions compared to *L. boryana*. This assumption is supported by the dithionite dependence of nitrogenase activity in CN strains but not *L. boryana* (Fig. 2). Nitrogenase activity of CN strains was observed only in the presence of dithionite, in contrast to the relatively high nitrogenase activity of *L. boryana* even in the absence of dithionite (Fig. 2A). In *Anabaena* sp. PCC 7120 (*Anabaena* 7120), the flavodiiron protein (encoded by *flv3B*) catalyzing oxygen reduction to water is essential for nitrogenase activity in heterocysts under aerobic conditions^35^. While *Synechocystis* 6803 has four Flv isoforms, they appear to be specialized for oxygen protection of PSII (Flv2 and Flv4) and PSI (Flv1 and Flv3). Very low nitrogenase activity in the CN strains suggests that the basal activities of the four Flv isoforms are insufficient for protection of nitrogenase from oxygen. It is possible, however, that overexpression of diazotrophic cyanobacterial *flv* genes such as *flv3B* may protect nitrogenase from oxygen, resulting in higher nitrogenase activity.

The *mop* gene encodes a small 7-kDa protein referred to as molbindin that is implicated in cellular storage and homeostasis of Mo^36,37^. Semi-quantification of *nif* gene cluster transcription levels^29^ indicated that *mop* is expressed in nitrogen-deficient conditions independent of oxygen level and its transcript is detected even in the absence of CnfR (*∆cnfR* mutant), in contrast to *nif* genes, which are expressed exclusively under nitrogen-deficient and micro-oxic (nitrogen fixation) conditions. Thus, the *mop* gene was not included in the first transformant CN1. However, as a constituent of FeMo-co, molybdenum is required for active nitrogenase. The *mop* gene is adjacent to the *modABC* genes encoding the Mo transporter in the *nif* gene cluster of *L. boryana*. No *mop* ortholog is found in the *Synechocystis* 6803 genome, while there are *modABC* orthologs (*sll0738* as *modA* and *sll0739* as *modBC*). Given that the *mop* gene may play an important role in nitrogenase assembly, we isolated a second transformant, CN2, including the *mop* gene in addition to the gene repertory of CN1. However, the acetylene reduction activity of CN2 was even lower than that of CN1 (Fig. 2B, p < 0.05), suggesting that *mop* does not enhance nitrogenase activity under the conditions examined in the heterologous host *Synechocystis* 6803.

The heterocystous cyanobacterium *Anabaena* 7120 has three *cox* gene clusters (*cox1*, *cox2*, and *cox3*), each encoding three subunits for COX. In contrast to constitutive expression of *cox1* genes in vegetative cells, *cox2* and *cox3* clusters are expressed specifically in heterocysts in response to nitrogen-deficient conditions^38^. A targeted mutant in which both *cox2* and *cox3* gene clusters were disrupted lost diazotrophic growth ability and demonstrated severely reduced nitrogenase activity (8%), indicating a critical contribution of heterocyst-specific *cox* genes in nitrogen fixation. *L. boryana* also harbors three *cox* gene clusters, one of which, *coxB2-coxA2-coxC2*, is contained within the *nif* gene cluster^29^. We isolated the third transformant CN3, in which the *coxB2-coxA2-coxC2* gene cluster was artificially connected to the truncated *nifB* promoter. We expected that these introduced *cox* genes would contribute to the protection of nitrogenase against oxygen by enhancement of COX activity. However, CN3 exhibited comparable or even slightly lower nitrogenase activity to that of CN1 (Fig. 2B, p = 0.38). To estimate the effect of the *cox* genes in CN3, we measured respiratory (oxygen consumption) activity (Supplementary Fig. 7). However, the respiratory activity of CN3 was about the same levels as WT, CN1, and CN2, and the expected effect of the *cox* genes on nitrogenase activity was not observed. While the truncated *nifB* promoter covers all conserved motifs (motifs I to IX) for recognition by CnfR^30^, the expression level might not be high enough for overexpression of the *cox* genes to show a significantly high COX activity. Thus, further improvement is required for higher expression of the *cox* genes in CN3.

Various strategies have been proposed for transplantation of nitrogen fixing ability to crops. Introduction of *nif* genes directly into the crop genome to express active nitrogenase is one promising approach. Current basic researches on this approach use *E. coli*, yeast, and tobacco as models. Based on the functional expression of *Klebsiella pneumonia nif* genes in *E. coli*^39,40^, Dixon and Wang specified a minimal set of 9 *nif* genes from *Paenibacillus* required for expression of active nitrogenase in *E. coli*^16^. They also identified 10 genes from *Azotobacter vinelandii* that are sufficient for active FeFe-type nitrogenase expression in *E. coli*^5^. Recently, they demonstrated that plant ferredoxins and their reductase systems are competent to donate electrons to MoFe- and FeFe-nitrogenases in the *E. coli* system^18^. Mitochondria and chloroplasts have been proposed as intracellular compartments to accommodate nitrogenase in the plant cell^14,17^. To this end, Rubio’s group reported successful expression of active Fe protein in yeast mitochondria^41^, but expression of active MoFe protein in yeast mitochondria remains difficult even when using extensive synthetic approaches^42^. In tobacco, 16 *nif* genes from *K. pneumoniae* were expressed as fusion proteins with mitochondrial targeting signals under the control of the 35 S promoter, and most were successfully localized in mitochondria. However, some gene products, including NifD, NifE, and NifQ, were difficult to detect immunologically in tobacco cells^43^. As an attempt to express nitrogenase in chloroplasts, Researchers at Monsanto Company isolated transgenic tobacco plants harboring the *nifH* and *nifM* genes in the chloroplast genome, and a very tiny but significant Fe protein activity was detected^44^.

In this study, we present the first successful functional expression of nitrogenase in an oxygenic photosynthetic organism, *Synechocystis* 6803. *Synechocystis* 6803 has several potential advantages as a model. First, *nifM* and *nifQ* are not included in the CN strains because orthologs are not found in the genomes of any diazotrophic cyanobacteria, including *L. boryana*^29,45^. This *nifM*- and *nifQ*-independent nitrogen fixation is a feature of cyanobacteria and is not found in heterotrophic diazotrophs such as *A. vinelandii* and *K. pneumonia*. Second, cyanobacteria share a common origin with plant chloroplasts, so endogenous ferredoxins and their reducing systems can be used as the electron donors for nitrogenase^18^. Third, nitrogenase coexists with oxygenic photosynthesis in non-heterocystous nitrogen-fixing cyanobacteria such as *L. boryana* and *Cyanothece*. Identifying the molecular mechanisms allowing nitrogen fixation and photosynthesis (i.e., solving the “oxygen paradox”) may enable functional expression of nitrogenase in the chloroplasts by simply coexpressing the underlying genes with *nif* genes. Transcriptional activation of *nif* genes by CnfR in response to micro-oxic conditions may be a critical step of this process.

The CN strains isolated in this study provide valuable platforms for investigating environmental factors and unknown genetic mechanisms necessary for functional expression of nitrogenase in chloroplasts.

## Materials and Methods

### Cyanobacterial strains and cultivation conditions

*Synechocystis* sp. PCC 6803 strain YF^46^ was used as the recipient of the *nif* genes from *Leptolyngbya boryana* strain *dg5*. We used BG-11 media for all strains. Instead of ferric ammonium citrate (6 mg L^−1^), NaNO_3_ (1.5 g L^−1^), and Co(NO_3_)_2_-6H_2_O (0.0494 g L^−1^ in Trace metal mix A5 + Co), a ferric citrate mixture (citric acid 1.2 mg, ferric citrate 1.2 mg and Na_2_EDTA 0.2 mg), NaCl (1.03 g L^−1^), and CoCl_2_ (0.022 g L^−1^ in a modified Trace metal mix A5 + Co) were added to base media that contained no combined nitrogen (BG-11_0_; for nitrogen-depleted medium)^47^. For nitrogen-replete medium (BG-11), KNO_3_ (final, 15 mM) was added. All media contained 20 mM HEPES-KOH; pH 8.2 for *Synechocystis* 6803^34^ or pH 7.4 for *L. boryana*^48^. Bacto Agar (Becton, Dickinson and Company, Franklin Lakes, NJ) was used for solidification of media (1.5% (w/v)). For selection of transformants, kanamycin (15 µg ml^−1^), spectinomycin (15 µg ml^−1^), and chloramphenicol (10 µg ml^−1^) were added. Agar plates were cultivated in the light (50 µmol m^−2^ s^−1^) at 30 °C. For micro-oxic conditions, agar plates were incubated in an anaerobic jar (BBL GasPak anaerobic systems, BD Biosciences) with a sachet for producing anaerobic conditions (Gas Generating Kit Anaerobic System; Oxoid, Basingstoke, Hants, UK)^46^. The “micro-oxic” conditions in this work means that the gas phase in a jar is kept anaerobic while the cyanobacterial cells in the jar still evolve oxygen by photosynthesis^34^. The evolved oxygen is quickly consumed by reaction with hydrogen generated from the sachet in the presence of an active catalyst in the jar. For check of anaerobic conditions in the jar, dry anaerobic indicator strips (Dry Anaerobic Indicator Strips, BD Biosciences) were used. Strips (containing methylene blue) showing blue under aerobic conditions turned white within one hour when the jar was closed for anaerobic incubation. We confirmed that the color of strips was kept white during incubation. In addition, using a small portable oxygen monitor (OXY-2, Jikco, Tokyo, Japan) we have confirmed that the oxygen level in the jar became 0% during induction.

### Plasmid construction

Plasmid vectors, PCR templates, PCR primers, and restriction enzymes used for plasmid constructions are summarized in Supplementary Figures 1 and 2, and Supplementary Tables 1 and 2. PCR fragments were amplified by KOD FX Neo DNA polymerase (Toyobo, Osaka, Japan), and purified from agarose gel using the Wizard SV Gel and PCR Clean-up System (Promega, Madison, WI). After digestion of PCR fragments and plasmids with restriction enzymes, DNA fragments were separated by agarose gel electrophoresis and purified by Wizard SV Gel and PCR Clean-up System. Ligation reactions were performed with the DNA Ligation Kit <Mighty Mix> (Takara, Shiga, Japan). *E. coli* JM109 strain competent cells were used for transformation. DNA sequences of the inserted fragments were confirmed by an ABI3100 sequencer (Applied Biosystems, Foster City, CA).

### Construction of pNSsacB2

The kanamycin resistance (Km^R^) cassette and the *sacB* gene were amplified from pRL271 (Accession number L05081; C. P. Wolk^49^,) and pYFC10^50^, and fused to a single 4.0-kb fragment by overlap PCR (Supplementary Table 1). This fusion fragment was cloned into the BamHI sites of pUC19 to form pUCK19sacB2. The upstream (*sll2030*) and downstream (*slr2031-ssr1880*) fragments of chromosomal neutral site III were amplified from genomic DNA of *Synechocystis* 6803 and cloned sequentially into the XbaI-XhoI and SacI sites of pUCK19sacB2, respectively, to form pNSsacB2. It should be noted that the *sacB* gene was used just temporarily in these initial plasmids (pUCK19sacB2 and pNSsacB2) and never in the later plasmids.

### Construction of pSN1KF

The single Km^R^ gene (amplified from pNSsacB2) was introduced into the XhoI and KpnI sites of pNSsacB2 to form pNSKmF, with the Km^R^ gene in the same direction as *slr2030-slr2031*. During this process, the *sacB*-Km^R^ gene was replaced with the Km^R^ gene. A 4.17-kb chromosomal fragment containing *fdxN*, *feoA*, *fdxH*, *hesB*, *hesA*, *nifW*, *orf70*, *orf115*, and partial *nifX* amplified from genomic DNA of *L. boryana dg5* was cloned into the XhoI site of pNSKmF to form pSN1KF (Supplementary Figs 1 and 2).

### Construction of pSN2SF

pNSsacB2 was digested with XhoI and KpnI, and the spectinomycin resistance (Sp^R^) fragment amplified from p6803NS2S1^30^ was ligated to form pNSspeF, with the Sp^R^ gene in the same direction as *slr2030-slr2031*. A 5.53-kb chromosomal fragment containing *nifW*, *orf70*, *orf115*, *nifX*, *nifN*, *nifE*, *orf99*, *dpsA*, *orf84*, and partial *nifP* amplified from the *L. boryana dg5* genome was introduced into the SalI-XhoI sites to form pSN2SF.

### Construction of pSN3KR

pNSsacB2 was digested with KpnI and self-ligated to form pNSKmR, with the Km^R^ gene in the direction opposite to *slr2031-slr2031*. A 5.41-kb chromosomal fragment containing partial *nifE*, *orf90*, *dpsA*, *orf84*, *nifP*, *nifB*, and partial *fdxN* was cloned into the SalI-XhoI sites to form pNS3KR.

### Construction of pSN4SFc

The fourth plasmid pSN4SF was constructed from pNSspeF by the introduction of a 5.53-kb fragment containing partial *nifB*, *fdxN*, *nifS*, *nifU*, *nifH* and partial *nifD*. However, no colonies carrying pNS4SF was obtained. We suspected that the partial *nifB* (the leftmost gene of the fragment) was unexpectedly expressed by read-through from the *lac* promoter of the parental plasmid pUC19 even in the absence of IPTG, resulting in a severe negative effect on growth of *E. coli*. To alleviate this probable negative effect, a 663-bp fragment (partial *modB* and *modC* genes) was introduced into the SalI-XhoI sites of pNSspeF to form pNSspeFc. The 5.53-kb fragment was successfully cloned into the XhoI site of pNSspeFc to form pSN4SFc.

### Construction of pSN5KR

A 5.80-kb chromosomal fragment containing partial *nifH*, *nifD*, *nifK*, *nifV*, *nifZ*, and *nifT* was introduced into the SalI-XhoI sites to form pSN5KR.

### Construction of pExCnfR5

The Km^R^ gene of pExCnfR4^30^ was removed by SacI and SalI digestion and the SacI-XhoI Sp^R^ gene from p6803NS2S1^30^ was ligated to form pExCnfR5.

### Construction of pSN6mop

A Cm^R^ cartridge (EcoRV-HincII) derived from pBR325 (Accession, L08855;^51^) was cloned into pBluescript II SK+ (Accession, X52328;^52^) to form pBSC9. The Cm^R^ cartridge was excised as the BamHI-BclI fragment, and inserted into pUC19 to form pUC19Cm2. The *slr2030* fragment was inserted into the SalI site of pUC19Cm2 to form pUCC6803L. A 1.77-kb chromosomal fragment containing *mop*, *fdxB*, *feoA*, *fdxH*, *hesB*, and partial *hesA* was inserted into the SacI-BamHI sites of pUCC6803L to form pSN6mop.

### Construction of pSN6cox

A *nifB* promoter fragment (484-bp) was inserted into the BamHI site to form pSN6Emp. Then, a 3.83-kb chromosomal fragment containing *coxB2*, *coxA2*, *coxC2*, and partial *orf159* was inserted into the BamHI site of pSN6Emp to form pSN6cox.

### Transformation of Synechocystis 6803

Transformation of *Synechocystis* 6803 was performed as previously described^30^. The 20-kb *nif* gene fragment, the central part of the 50-kb *nif* gene cluster of *L. boryana*, was divided into 5 subfragments (each 4–5 kb) and sequentially inserted into the genome neutral site III by homologous recombination. The first plasmid pSN1KF carried a 4.17-kb *nif* subfragment (Fragment I) flanked by two homologous fragments of *slr2030* and *slr2031*, and the Km^R^ gene was used as the selection marker. The 1^st^ transformant SN1 was isolated from WT by transformation with pSN1KF. The second plasmid pNS2SF carried a 5.53-kb *nif* subfragment (Fragment II) in which the left 1-kb was used as the left homologous sequence. The Sp^R^ gene was included as another selection marker. The 2^nd^ transformant SN2 was isolated from SN1 by transformation with pNS2SF. Thus, by utilizing the selection markers (Km^R^ and Sp^R^) alternately, the 5^th^ transformant SN5 was isolated (Supplementary Figs 1 and 2). The *cnfR* gene under the control of the *trc* promoter was introduced into another genome neutral site I of SN5 to isolate transformant CN1 carrying the 20.8-kb (20,763 bp) *nif* gene cluster and the *trc*-driven *cnfR* gene with the selective markers Km^R^ and Sp^R^. Using CN1 as the host strain, the other transformants CN2 and CN3 were isolated by the introduction of pSN6mop and pSN6cox, respectively. Completion of the segregation was confirmed in all mutants by genomic PCR.

### Growth under nitrogen fixing conditions

CN strains grown under aerobic and nitrogen-replete conditions were suspended in distilled water. After adjustment of cell density (OD_730_), aliquots (5.0 µl) of the suspensions were spotted onto BG-11_0_ agar plates. The plates were incubated under aerobic or micro-oxic conditions in the light (50 µmol m^−2^ s^−1^) at 30 °C.

### Assay of nitrogenase activity

Induction of the *nif* genes and nitrogenase assay were essentially the same as described previously^29^. Cells were grown on BG-11 agar plates under light conditions (50 µmol m^−2^ s^−1^) at 30 °C for 3 days as a pre-culture. The cells were collected in distilled water adjusted to OD_730_ 9.2, and 250 µl of the cell suspension was spread uniformly to form a 4-cm diameter circle on a BG-11_0_ agar plate. The agar plate was incubated in an anaerobic jar under light conditions (50 µmol m^−2^ s^−1^) at 30 °C for 14 h. The cells were harvested with 1.5 ml of BG-11_0_ liquid medium and an aliquot (1.0 ml) of the suspension was transferred into a 5-ml glass vial (V-5A, Nichiden-rika glass, Kobe, Japan) in the anaerobic chamber. To stimulate *in vivo* nitrogenase activity dithionite was added to the suspension (final concentration: 5 mM^31^). The glass vials were sparged with a gas mixture (10% (vol/vol) acetylene in argon as standard gas; Japan Fine Products, Kawasaki, Japan) for 45 sec. The glass vials were incubated for 2-5 h under illumination (50 µmol m^−2^ s^−1^) at 30 °C with stirring. The upper gas phase (500 µl) was analyzed by a gas chromatograph (GC-2014AF, Shimazdu, Kyoto, Japan) equipped with a Porapak N column (0.3 m × 3 mm, Shinwa Chemical Industrials, Kyoto, Japan) under isothermal conditions (40 °C). Elusion of ethylene (at 0.64 min in this condition) was detected by an FID detector. After the assay of ethylene formation assay, the cells were collected for measurement of optical density at 730 nm (OD_730_) with a spectrophotometer (model UV1700 or UV1600; Shimadzu). Chlorophyll content of the suspension (500 µl) was also determined as described^53^.

### Determination of the relationship between OD_730_ and CDW

CDWs of cell suspensions of *Synechocystis* 6803 and *L. boryana* with various OD_730_ values (0 to 50) were measured as described in Kato *et al*.^54^, and a linear relationship between CDW and OD_730_ was determined for each species (Supplementary Fig. 3). CDW was estimated from OD_730_ based on the linear relationship between them.

### Determination of respiratory and photosynthesis activities with an oxygen electrode

Cells of WT (strain YF), CN1, CN2 and CN3 were subjected to induction of the *nif* genes as described above. The cells harvested with 1.5 ml of BG-11_0_ liquid medium were diluted three-fold with BG-11_0_, an aliquot (0.99 ml) of the cell suspension was used for measurement of oxygen consumption in the dark and oxygen evolution in the light with a Clark-type oxygen electrode (DW1, Hansatech, Norfolk, UK). Sodium bicarbonate (10 µl of 1 M, final 10 mM) was added just before measurement.

### Western blot analysis

Cells were grown on BG-11 agar plates under light conditions (50 µmol m^−2^ s^−1^) at 30 °C for 3 days as a pre-culture. The cells were collected in distilled water and adjusted to OD_730_ 15.6. Aliquots of cell suspension (500 µl) were spread uniformly to form 4-cm diameter circles on BG-11_0_ agar plates. Plates were then incubated in an anaerobic jar under light conditions (50 µmol m^−2^ s^−1^) at 30 °C for 14 h. Induced cells in the anaerobic chamber were harvested in 700 µl of protein extraction buffer (50 mM HEPES-KOH; pH 7.5, 10 mM MgCl_2_) and an aliquot (500 µl) was transferred into a 1.5-ml micro-centrifuge tube containing glass beads (100 mg glass beads; 150–212 microns, Sigma, St. Louis, MO). The cells were disrupted by vigorous shaking (Setting LA; “BugCrasher” GM01, Taitec, Saitama, Japan) at 4 °C for 1 h. The resultant homogenates were centrifuged at 13,600 × g for 3 min at 4 °C to prepare the supernatant fraction. Protein concentration was determined by Bradford assay (Protein Assay, Bio-Rad, Hercules, CA) with bovine serum albumin as the standard. Western blot analysis was carried out as described previously^55^. The specific protein bands were visualized by a lumino-image analyzer (LAS-3000mini, Fujifilm, Tokyo, Japan). The obtained images were converted to tiff images by Multi Gauge software (Fujifilm). The signal intensities of the NifH, NifD, and NifK proteins on the tiff images were quantified by ImageJ ver 1.50i.

The NifH, NifD, and NifK proteins were detected by specific antisera prepared against *L. boryana* NifH, NifD, and NifK proteins as Strep-fusion proteins (Scrum, Tokyo, Japan). These proteins were expressed in *E. coli* and purified by Strep-Tactin affinity column.

## Electronic supplementary material

Supplementary Information
