# Supplementary material for: Functional expression of an oxygen-labile nitrogenase in an oxygenic photosynthetic organism
Source: Sci Rep. 2018 May 9;8:7380. doi: 10.1038/s41598-018-25396-7 (PMC5943405; doi:10.1038/s41598-018-25396-7)
Supplement: Supplementary file 1 — Supplementary Information [file 41598_2018_25396_MOESM1_ESM.docx]

**Supplemental Data**

**Functional expression of an oxygen-labile nitrogenase in an oxygenic photosynthetic organism**

Ryoma Tsujimoto^a^, Hiroya Kotani^a^, Konomi Yokomizo^a^, Hisanori Yamakawa^a^, Aoi Nonaka^b^, and Yuichi Fujita^a^*

^a^Graduate School of Bioagricultural Sciences, Nagoya University; ^b^School of Agricultural Sciences, Nagoya University

*Corresponding Author: Yuichi Fujita

Contents:

**Supplementary Figure 1** p. 2, Scheme of plasmid construction

**Supplementary Figure 2** p. 3, Detailed transformation procedure

**Supplementary Figure 3** p. 4, Determination of relationship between OD_730_ and CDW

**Supplementary Figure 4** p. 5, Gas chromatogram of ethylene formation

**Supplementary Figure 5** p. 6, Nitrogenase activity on per-chlorophyll and per-OD_730_ bases

**Supplementary Figure 6** p. 7, Growth comparison under diazotrophic conditions

**Supplementary Figure 7** p. 8, Oxygen evolution and consumption of CN1, CN2 and CN3

**Supplementary Table 1** p. 9-10, Plasmid construction and oligonucleotide list

**Supplementary Table 2** p. 11, Plasmids used for isolation of transformants


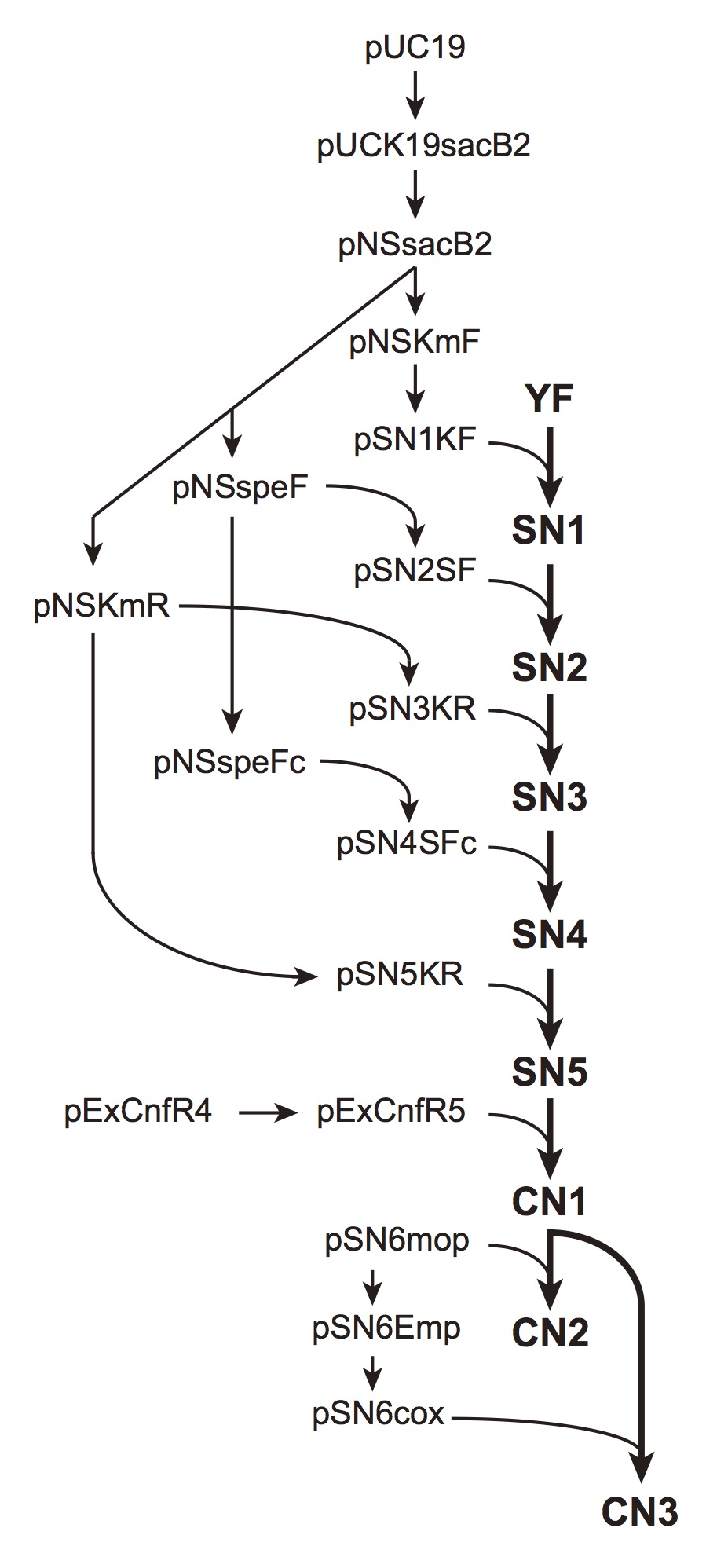


**Supplementary Figure 1.** Scheme of plasmid construction (thin lines) and isolation of CN1, CN2 and CN3 from YF (thick lines).

**
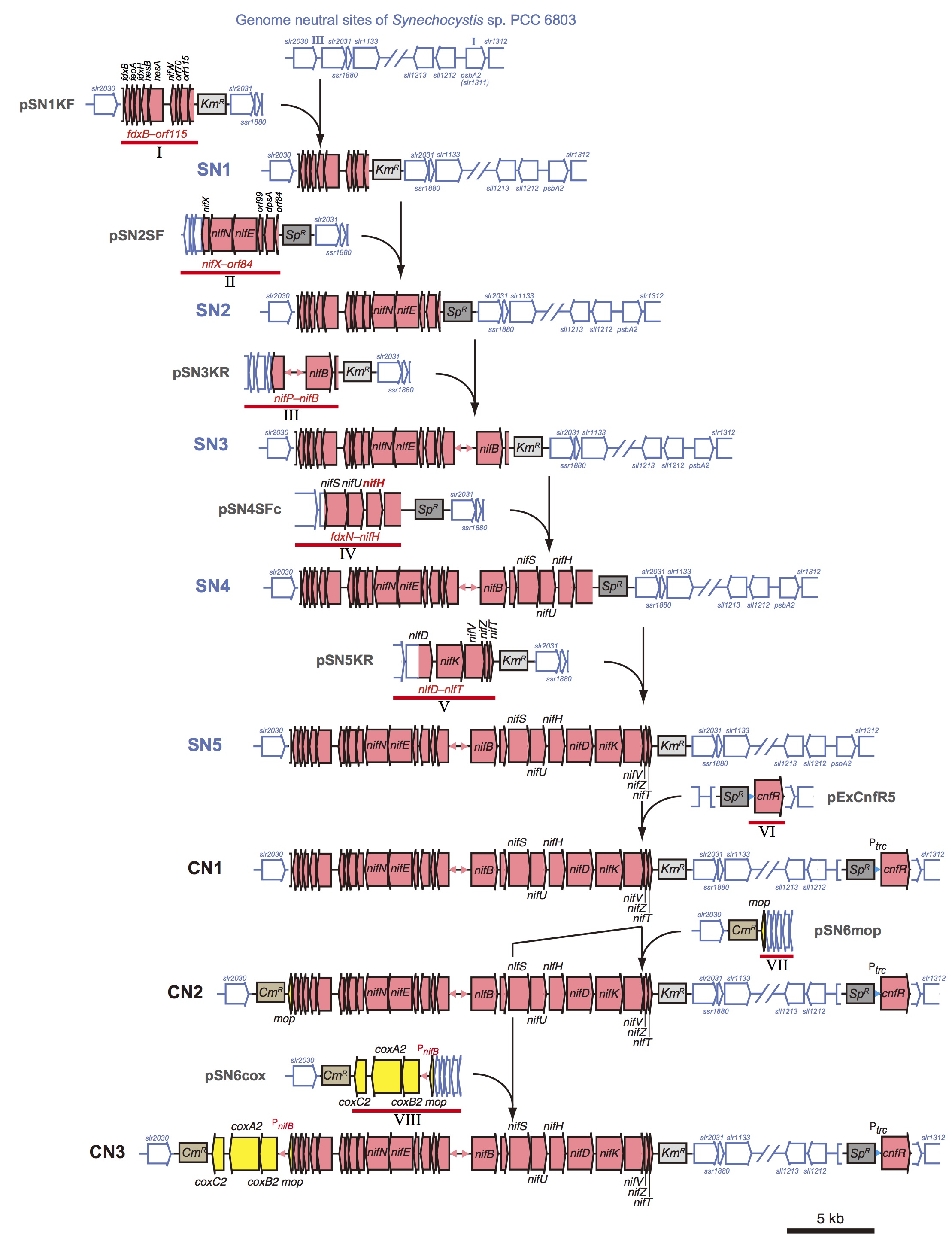
**

**Supplementary Figure 2.** Detailed transformation procedures for CN1, CN2, and CN3.


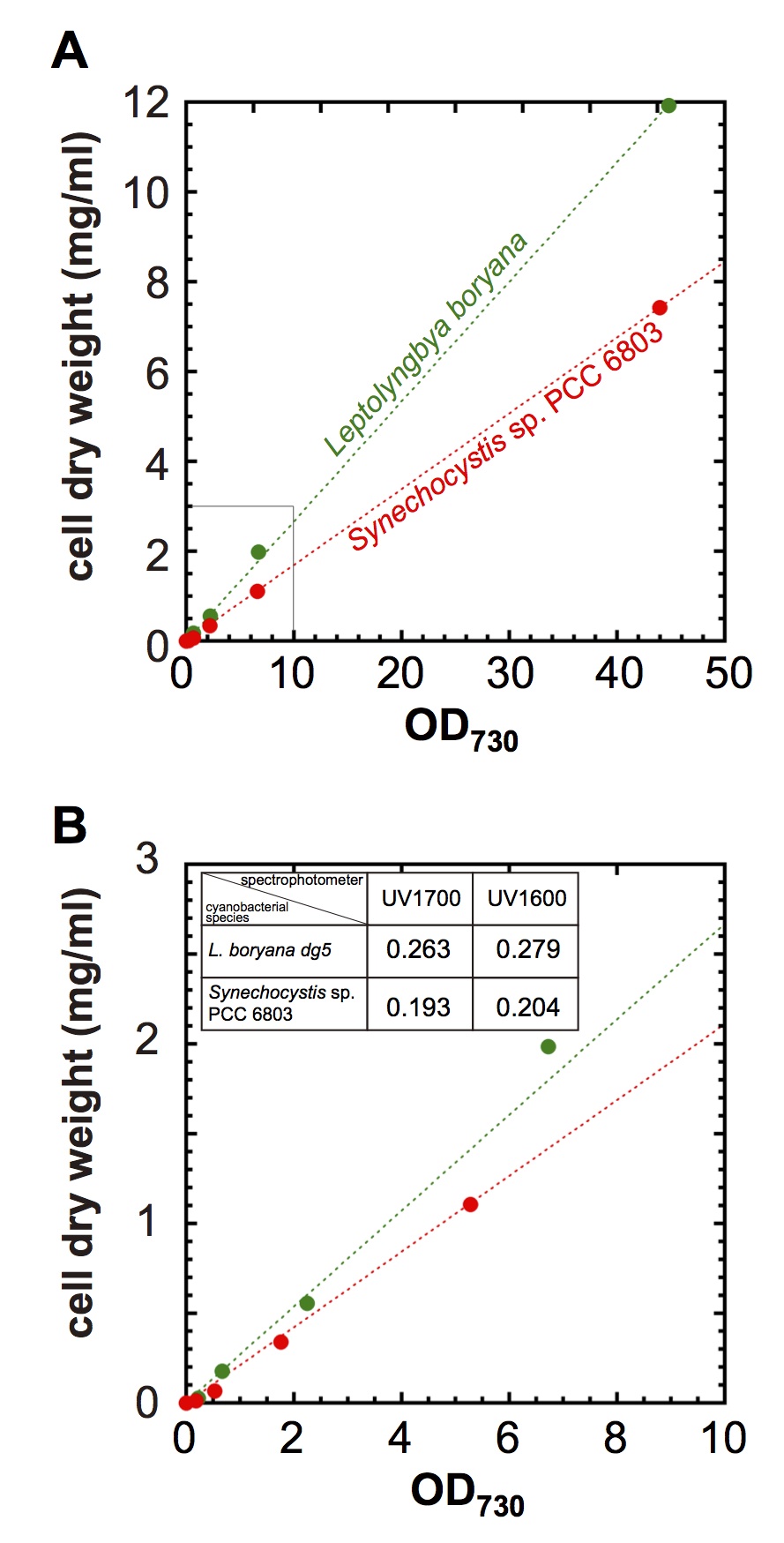


**Supplementary Figure 3.**  Determination of linear relationship between OD_730_ and CDW in *L. boryana* and *Synechocystis* 6803. CDWs were determined for cell suspensions of *L. boryana* (green) and *Synechocystis* 6803 (red) with various OD_730_ values of 0 to 50 (A) and 0 to 10 (B). Panel B is an enlarged graph of panel A (shown by a small square in panel A). These plots are typical set of triplicates, and OD_730_ values in this figure were determined by a spectrophotometer UV1600 (Shimadzu). Proportional constants were determined from these relationships (Inset table of panel B), and the OD_730_ values (Supplementary Figure 5B) were converted to the CDW values (Fig. 2A).


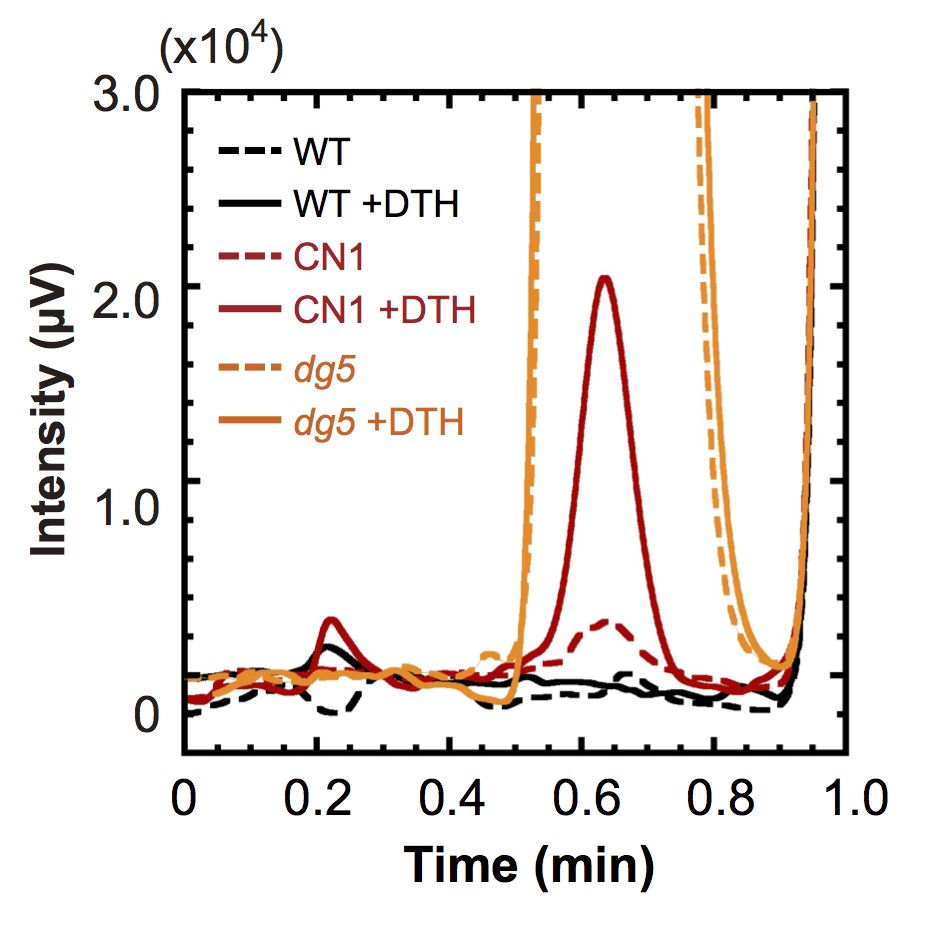


**Supplementary Figure 4.** Gas chromatograms of ethylene formation in the wild type (WT, black), CN1 (red), and *L. boryana dg5* (orange) in the presence (solid lines) and absence (dashed lines) of dithionite. This is a typical chromatogram for determination of ethylene formation in Figure 2A.


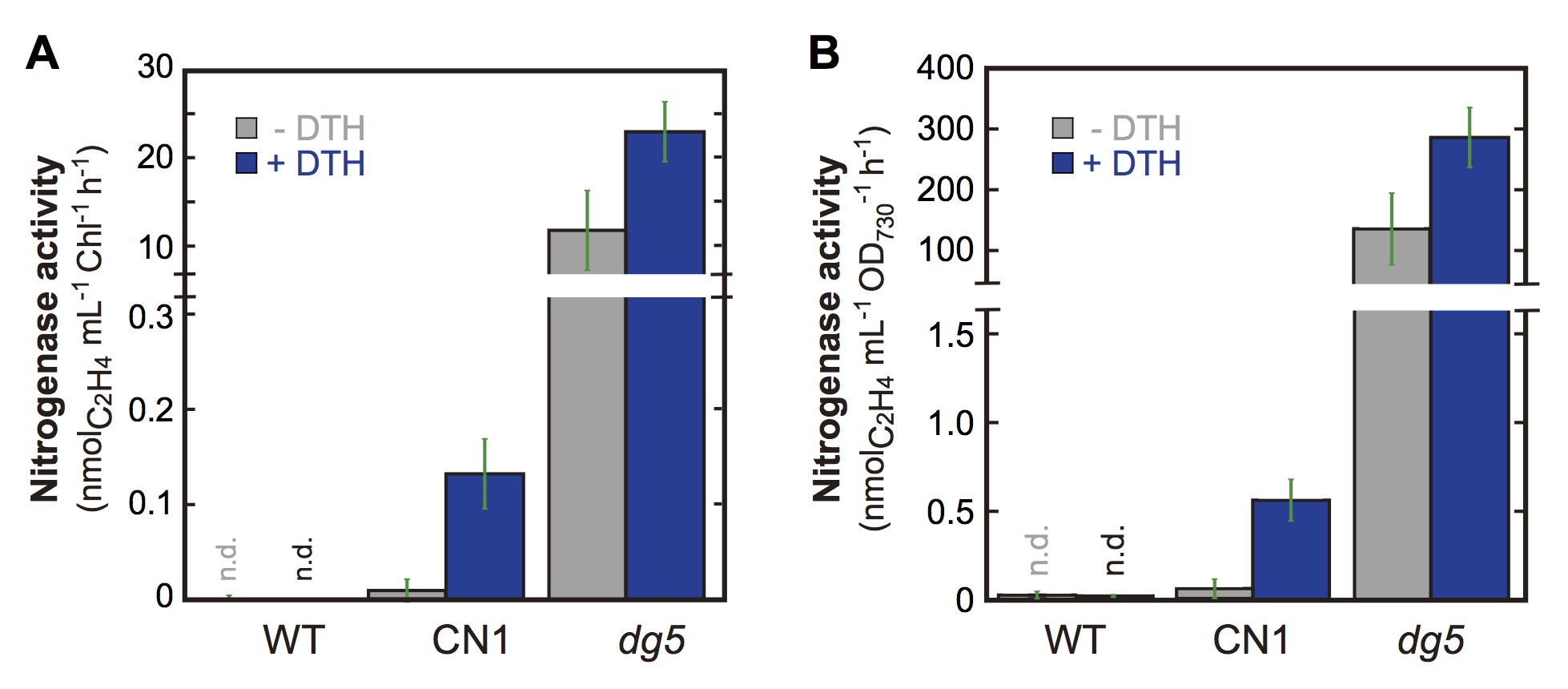


**Supplementary Figure 5.** Nitrogenase activity of CN1 compared to *Synechocystis* 6803 WT and *L. boryana dg5* on a per-chlorophyll basis (A) and a per-OD_730_ basis (B). Ethylene formation was estimated in the presence (blue) and absence (gray) of dithionite. Error bars (green) represent standard deviations (the same experiments as Fig. 2A). n.d. denotes “not detected”.


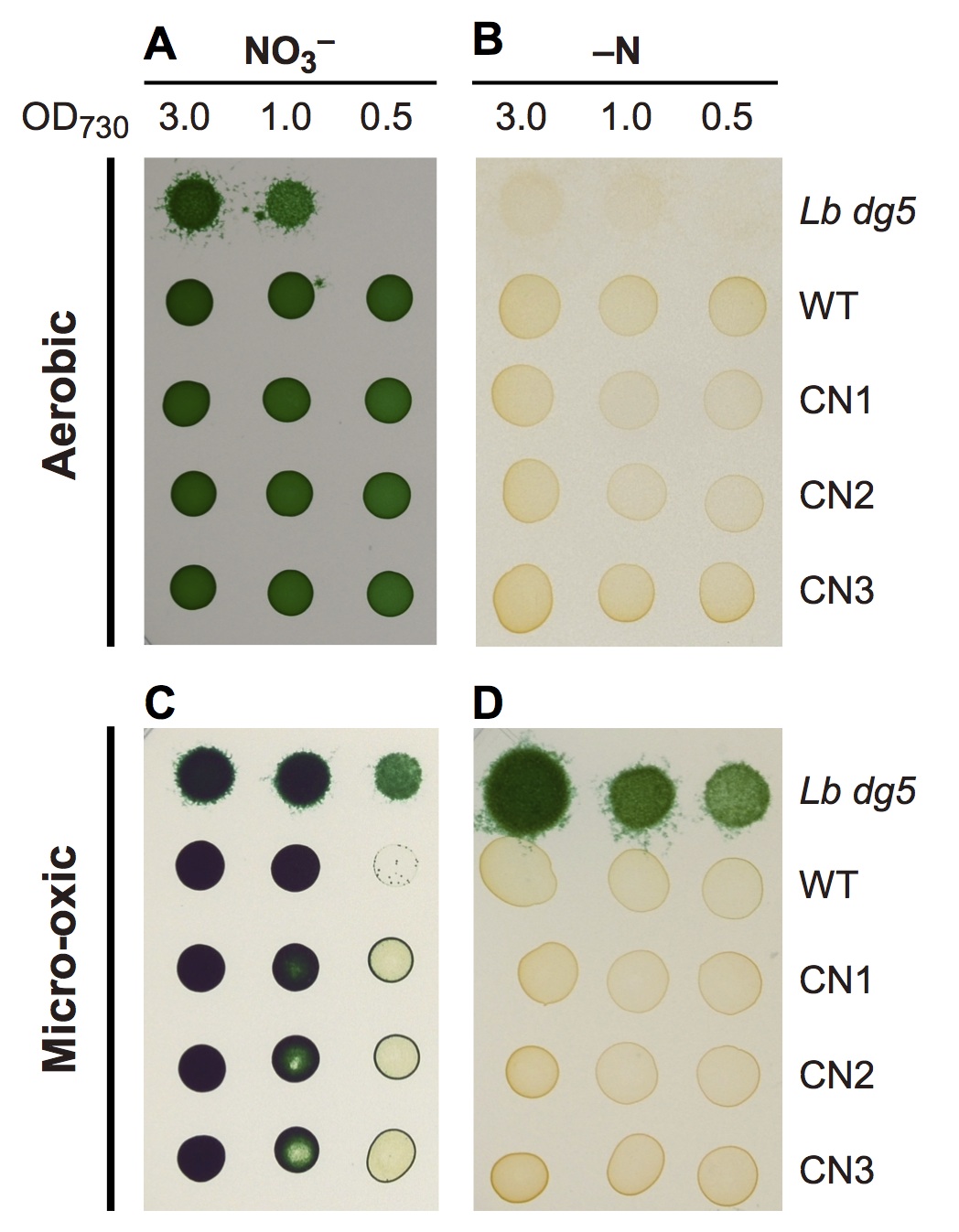


**Supplementary Figure 6.** Growth comparison of *Synechocystis* 6803 (Wild type), CN1, CN2, CN3, and *L. boryana dg5* (*Lb dg5*) on BG-11 (**A**, and **C**) and BG-11_0_ (**B**, and **D**) agar plates under aerobic (**A** and **B**) and micro-oxic conditions (**C**, and **D**) at 30 ºC. Incubation times were 7 days (**A**), 17 days (**C**) and 23 days (**B** and **D**). Aliquots (5 µl) of suspensions adjusted to OD_730_ 3.0, 1.0, and 0.5 were spotted onto the agar plates.


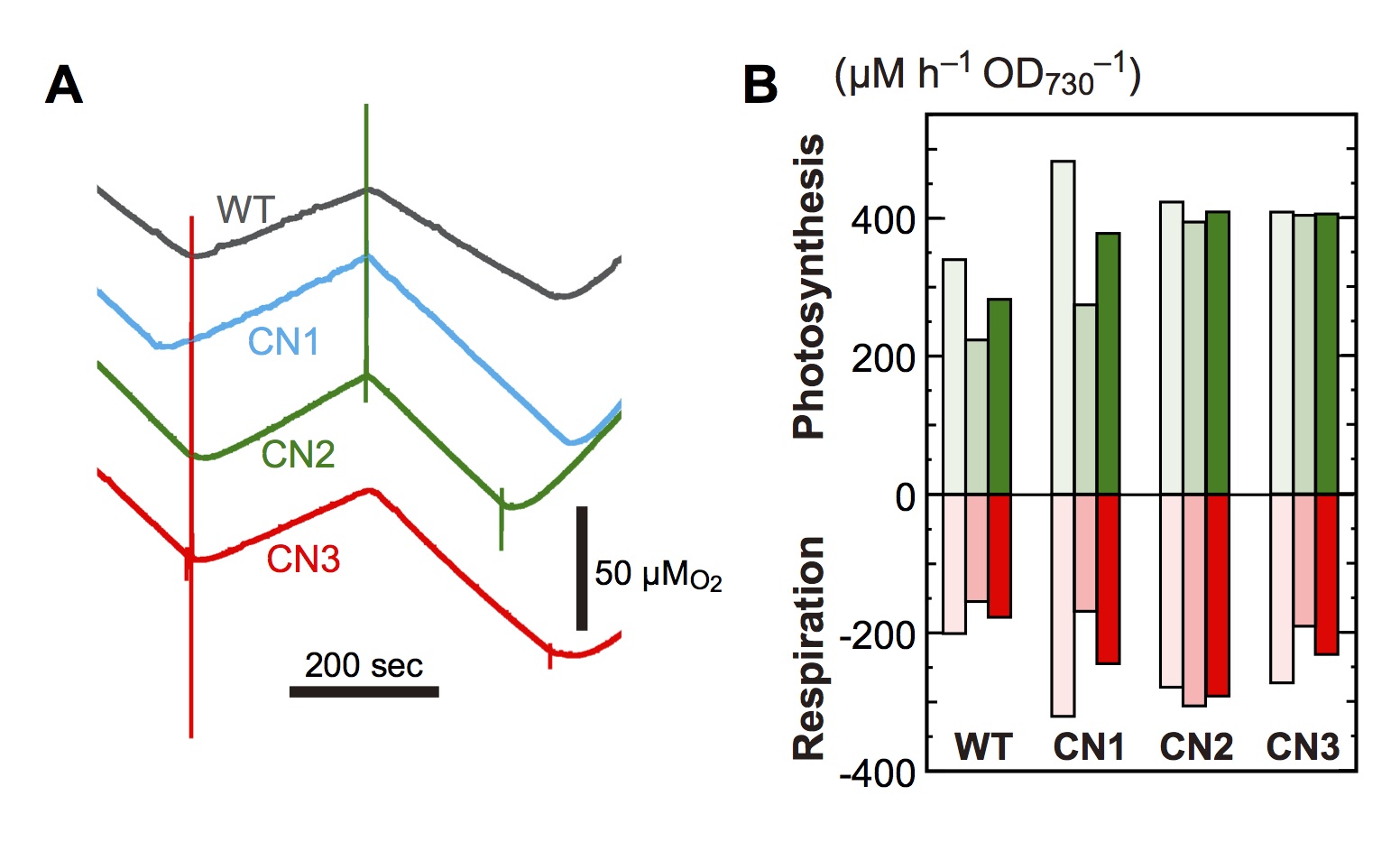


**Supplementary Figure 7.** Oxygen evolution and consumption of *Synechocystis* 6803 (WT), CN1, CN2, and CN3. Cells induced on BG-11_0_ plates were collected, and the cell density was adjusted to OD_730_ 1 to 2 and an aliquot (990 µl) was set in a cuvette of a Clark-type oxygen electrode (30ºC). After dark incubation for 3 min, cells was illuminated with light (50 µmol m^–2^ s^–1^) for 4 min followed by dark incubation for 3 to 4 min (A, These profiles are typical traces of the measurements in duplicates). Respiratory activity was estimated from oxygen consumption rate in the 4-min dark incubation. Photosynthesis activity was estimated from oxygen evolution rate in the 4-min light incubation, which was corrected by the respiratory activity. Two bars in pale green and pale red show individual activities of photosynthesis and respiration, respectively, in the duplicates, and green and red bars indicate the average values of photosynthesis and respiration, respectively.

**Supplementary Table 1.** Plasmid construction and oligonucleotide list

| Plasmid^1^ | Origin^2^ | Enz.^3^ | Insert^4^ | Primer | | Primer sequence (5'-3')^5^ | Enz.^6^ | Description^7^ |
| --- | --- | --- | --- | --- | --- | --- | --- | --- |
| pUCK19sacB2 | pUC19 | Bm | PCRoe ^8^  (pRL271, pYFC10) | sacBf1 | | TAGGATCCTGCAGGTCGACTCTAGCTAG | Bm | *sacB*-Km^R^ fusion |
|  |  |  |  | sacBr1 | | CTTGCGGCAGCGTGAAGCTTACGGAAGTCGCTGTCGTTCT |  |  |
|  |  |  |  | neof1 | | AGAACGACAGCGACTTCCGTAAGCTTCACGCTGCCGCAAG |  |  |
|  |  |  |  | neor1 | | AAGGATCCGGACCACGCTATCTGTGCAA |  |  |
| pNSsacB1 | pUCK19sacB2 | Xb, Xh | PCR (S6803G) | sll1910-f2XbaI | | GAAGGGAAGCGTCTAGAATTGTCGAGG | Xb, Xh | Upstream region of the neutral site |
|  |  |  |  | slr2030-r1XhoI | | CTACAACTCGAGCCTATTGAACCAGC |  |  |
| pNSsacB2 | pNSsacB1 | Sa | PCR (S6803G) | slr2031-f3SacI | | TTGTTGAGCTCCCACTTCTCCGGTG | Sa | Downstream region of the neutral site |
|  |  |  |  | slr1133-r2SacI | | GAAATTACCGAGCTCGGTATTCCTGG |  |  |
| pNSkmF | pNSsacB2 | Kp, Xh | PCR (pNSsacB2) | KmR-f2XhoI | | CCACCGATCTCGAGAACGACAGC | Kp, Xh | Kanamycin resistance cassette |
|  |  |  |  | KmR-r2KpnI | | GGTTCAATAGGTACCGAGTGGGTG |  |  |
| pNSkmR ^9^ | pNSsacB2 | Kp | None |  | |  |  |  |
| pNSspeF | pNSsacB2 | Kp, Xh | PCR (p6803NS2S1) | SpeR-f2XhoI | | GTACCAAGCTCTCGAGTAACATCAAG | Kp, Xh | Spectinomycin resistance cassette |
|  |  |  |  | SpeR-r3KpnI | | GATTTAAAGCTCGGTACCAACTATTGC |  |  |
| pNSspeFc | pNSspeF | Sl, Xh | PCR (LboryG) | PbmodB-f2XhoI | | CGTTCTTGCTCGAGTTCGATTTCCAG | Xh | Part of the coding region of *modB* and *modC* |
|  |  |  |  | PbmodC-r3XhoI | | GTCATGCTTTTACTCGAGCCAGAACC |  |  |
| pSN1KF | pNSkmF | Xh | PCR (LboryG) | Pbmop-r5SalI | | AGCTAAGCCGTCGACTGAGGATTTCG | Sl, Xh | 1^st^ part of *nif* cluster (*fdxB*-*orf115*) |
|  |  |  |  | PbnifX-f1SacXho | | CTCATTTTGAGCTCGAGCCAGTAAGATTG |  |  |
| pSN2SF | pNSspeF | Sl, Xh | PCR (LboryG) | PbnifW-r3SalI | | GACAGAGAGTCGACACTCAGCGAAG | Sl, Xh | 2^nd^ part of *nif* cluster (*nifW*-*orf84*) |
|  |  |  |  | PbnifP-f2XhoI | | CGAACAGCTCGAGGAGCAACTGC |  |  |
| pSN3KR | pNSkmR | Sl | PCR (LboryG) | PbnifE-r5SalI | | GATCAGTGCAGTCGACAAGTCGAATAG | Sl, Xh | 3^rd^ part of *nif* cluster (*orf99*-*nifB*) |
|  |  |  |  | PbfdxN-r2XhoI | | GAATGGCTCCTCTCGAGCAAACTGG |  |  |
| pSN4SFc | pNSspeFc | Xh | PCR (LboryG) | PbnifB-f9XhoI | | TCACTATGCTCGAGATGCACGTTGC | Sl, Xh | 4^th^ part of *nif* cluster (*fdxN*-*nifH*) |
|  |  |  |  | PbnifD-r7SalI | | GCAATCTGAGTCGACCCGAAGAAG |  |  |
| pSN5KR | pNSkmR | Sl | PCR (LboryG) | PbnifH-f3SalI | | GACGTACTAGGTCGACGTTGTATGC | Sl | 5^th^ part of *nif* cluster (*nifD*-*nifT*) |
|  |  |  |  | PbnifT-r6SalI | | TGAACGAAGTCGACGTGAACGGGAC |  |  |
| pUC19Cm2 | pUC19 | Bm | pBSC9 ^10^ |  | |  | Bm, Bc |  |
| pUCC6803L | pUC19Cm2 | Sl | PCR (S6803G) | sll1910-f1SalI | | CGTTAGAATTGTCGACGTTTGCCATTG | Sl, Xh | Upstream region of the neutral site |
|  |  |  |  | slr2030-r1XhoI | | CTACAACTCGAGCCTATTGAACCAGC |  |  |
| pSN6mop | pUCC6803L | Sa, Bm | PCR (LboryG) | PbhesA-f1SacI | | CGATCGTTGGAGCTCACAACTTCGAG | Sa, Bm | *nif* cluster fragment (*mop*-*hesB*) |
|  |  |  |  | Pbmop-r3BamHI | | CCATGCTGAAGGATCCTATGAGACAG |  |  |
| pSN6Emp | pSN6mop | Bm | PCR (LboryG) | PbnifB-f31BglII | | CAACTAGATCTACAGAAAAACCCAAC | Bm, Bg | *nifB* promoter (478 bp) |
|  |  |  |  | PbnifB-r23BamHI | | CATGGATCCCATTGAATTTCGGAATAG |  |  |
| pSN6cox | pSeqNif6Emp | Bm | PCR (LboryG) | PbcoxB-f5BglII | | CTTGAAGCAGATCTATGAACCAAATTCC | Bm, Bg | Coding regions of *coxB2A2C2* |
|  |  |  |  | Pborf159-r3BamHI | | TTCAGGGATCCATCGTACCAGGAAAG |  |  |
| pExCnfR5^11^ | pExCnfR4^11^ | Sa, Sl | p6803NS2S1^11^ |  | |  | Sa, Xh | Spectinomycin resistance cassette |
|  |  |  |  |  |  | |  |  |

^1^ Name of the constructed plasmid.

^2^ Original plasmid used to construct the plasmid in the first row.

^3^ Restriction enzymes used to digest the original plasmid in the second row. Abbreviations are Bm, BamHI; Bc, BclI; Bg, BglII; Kp, KpnI; Sa, SacI; Sl, SalI; Xb, XbaI; and Xh, XhoI.

^4^ Methods used to prepare the insert DNA. The template DNA for PCR is shown in parentheses; S6803G, genomic DNA from *Synechocystis* 6803; LboryG, genomic DNA from *L. boryana*.

^5^ Primer sequence. Nucleotide sequences corresponding to the modified motifs are underlined.

^6^ Restriction enzymes used to digest the insert DNA. Abbreviations are the same as in annotation 3.

^7^ Description of the insert DNA.

^8^ The *sacB* gene was amplified by primers sacBf1 and sacBr1 with pRL271 (Black et al. 1993) as the template. The Km^R^ (*neo*) gene was amplified by primers neof1 and neor1 with pYFC10 (Fujita et al. 1992) as the template. The two DNA fragments were connected by overlap extension PCR (PCRoe).

^9^ pNSkmR was constructed by KpnI digestion to remove the *sacB* fragment (1,546 bp) followed by self-ligation.

^10^ The Cm^R^ gene cartridge (HincII-EcoRV) in pBR325 was cloned into the EcoRV site of pBluescript II SK+ to form pBSC9. The Cm^R^ gene was excised from pBSC9 by BamHI and BclI digestion for cloning into the BamHI site of pUC19.

^11^ pExCnfR5 was constructed by replacement of the Km^R^ gene in pExCnfR4 (Tsujimoto et al. 2016) with the Sp^R^ gene excised from p6803NS2S1 (Tsujitmoto et al. 2016).

**References**

Black, T.A., Cai, Y., & Wolk, C.P. Spatial expression and autoregulation of *hetR*, a gene involved in the control of heterocyst development in *Anabaena*. *Mol. Microbiol.* **9,** 77-84 (1993).

Fujita, Y., Takahashi, Y., Chuganji, M., & Matsubara, H. The *nifH*-like (*frxC*) gene is involved in the biosynthesis of chlorophyll in the filamentous cyanobacterium *Plectonema boryanum*. *Plant Cell Physiol.* **33,** 81-92 (1992).

Tsujimoto, R., Kamiya, N., & Fujita, Y. Identification of a *cis*-acting element in nitrogen fixation genes recognized by CnfR in the nonheterocystous nitrogen-fixing cyanobacterium *Leptolyngbya boryana*. *Mol. Microbiol.* **101,** 411-424 (2016).

**Supplementary Table 2.** Plasmids used for isolation of transformants

| Name | Relevant plasmid | Introduced genes^1^ | Homologous sequences | | Selective marker |
| --- | --- | --- | --- | --- | --- |
|  |  |  | Left arm^1^ | Right arm^1^ |  |
| SN1 | pSN1KF | *mop**-*fdxB*-*feoA*-*fdxH*-*hesB*-  *hesA*-*nifW*-*orf70*-*orf115*-*nifX** (4170) | *slr2030* (2049) | *slr2031*-*ssr1880*-  *slr1133** (1961) | Km^R^ |
| SN2 | pSN2SF | *nifX*-*nifN*-*nifE*-*orf99*-  *dpsA*-*orf84*-*nifP** (5530) | *nifW*-*orf70*-*orf115*-  *nifX** (1374) | *slr2031-ssr1880*-  *slr1133** (1961) | Sp^R^ |
| SN3 | pSN3KR | *nifP*-*nifB*-*fdxN** (5408) | *nifE**-*orf99*-*dpsA*-  *orf84-nifP** (1771) | *slr2031-ssr1880*-  *slr1133** (1961) | Km^R^ |
| SN4 | pSN4SFc | *nifB**-*fdxN-nifS-nifU-*  *nifH-nifD** (6073) | *nifB**-*fdxN** (1504) | *slr2031-ssr1880*-  *slr1133** (1961) | Sp^R^ |
| SN5 | pSN5KR | *nifD**-*nifK-nifV-nifZ-*  *nifT* (4236) | *nifH**- *nifD** (1570) | *slr2031-ssr1880*-  *slr1133** (1961) | Km^R^ |
| CN1 | pExCnfR5 | P*trc*-*cnfR* | *sll1212**-*psbA2** (1378) | *psbA2**-*slr1312** (1633) | Sp^R^ |
| CN2 | pSN6mop | *mop-fdxB-feoA-fdxH-*  *hesB-hesA** (1768) | *slr2030* (2049) | *fdxB-feoA-fdxH-*  *hesB-hesA** | Cm^R^ |
| CN3 | pSN6cox | *mop*-P*nifB*-*coxB2-coxA2-*  *coxC2* (4318) | *slr2030* (2049) | *mop-fdxB-feoA-*  *fdxH-hesB-hesA** (1768) | Cm^R^ |

^1^ Genes that partially introduced are indicated by asterisks. Length (bp) of the gene elements is shown in parentheses. P*trc* and P*nifB* indicate the promoters of *trc* and *nifB*.
